# Research Progress on the Application of Metal Porphyrin Electrochemical Sensors in the Detection of Phenolic Antioxidants in Food

**DOI:** 10.3390/polym17060789

**Published:** 2025-03-16

**Authors:** Liang Qu, Zhiyuan Lin, Feng Liu, Fanzhuo Kong, Yuyang Zhang, Xing Ni, Xue Zhang, Yani Zhao, Qiongya Lu, Bin Zou

**Affiliations:** 1 School of Food and Bioengineering, Wuhu Institute of Technology, Wuhu 241003, China; 101385@whit.edu.cn; 2 School of Food and Biological Engineering, Jiangsu University, Zhenjiang 212013, China; 17354948727@example.com (Z.L.); 2222218077@stmail.ujs.edu.cn (F.L.); 2222218033@stmail.ujs.edu.cn (F.K.); 2212218043@stmail.ujs.edu.cn (Y.Z.); 2212318069@stmail.ujs.edu.cn (X.N.); 2212318042@stmail.ujs.edu.cn (X.Z.); 2212418062@stmail.ujs.edu.cn (Y.Z.); 2222418088@stmail.ujs.edu.cn (Q.L.)

**Keywords:** phenolic antioxidants, electrochemical sensor, metalloporphyrin, multi-walled carbon nanotubes, covalent organic framework

## Abstract

This paper reviews the application of metal porphyrin electrochemical sensors in the detection of phenolic antioxidants in food, focusing on the latest progress and innovative applications in this field. Phenolic antioxidants are widely used in food and can effectively prolong the shelf life of food, but their excessive use may cause potential harm to human health, so the detection of their content is very important. In recent years, electrochemical analysis technology has gradually become an emerging method for quantitative detection of phenolic antioxidants due to its advantages of sensitivity, simplicity and high selectivity. As a new type of sensor, metal porphyrin electrochemical sensors have been widely used in the detection of phenolic antioxidants in food due to their excellent electrochemical performance and high selectivity. By modifying metal nanomaterials, the detection performance of these sensors has been significantly improved. This paper first introduces the basic concepts and physicochemical properties of phenolic antioxidants, analyzes their potential hazards and discusses relevant regulations and limit requirements. Then, the existing analysis methods of phenolic antioxidants are compared, and the development trend of traditional detection methods and new detection technologies is reviewed. Subsequently, the application progress of electrochemical sensors in the detection of phenolic antioxidants is discussed in depth, its working principle is expounded and the research results are summarized. Finally, the innovative applications of metalloporphyrins and their nanocomposites in electrochemical sensors are introduced in detail. The unique advantages of metalloporphyrins in the detection of phenolic antioxidants in food are highlighted, and the future development direction is laid out.

## 1. Introduction

### 1.1. Overview of Phenolic Antioxidants

The addition of antioxidants in foods can prevent the oxidative deterioration of foods and increase the shelf life of foods [1,2,3]. The proper use of antioxidants can not only bring good economic benefits to producers, but also bring safer food to consumers. Phenolic antioxidants are the most widely used in food. As a class of phenolic compounds with antioxidant properties, they prevent or inhibit the chain initiation and chain growth of free radical reactions by capturing free radicals, so as to achieve antioxidation [4,5,6]. At present, the common phenolic antioxidants in the food field can be divided into two categories: natural phenolic antioxidants (such as tocopherols) and synthetic phenolic antioxidants (such as tert-butylhydroquinone) [6,7,8,9]. Due to the difficulties in extraction and poor stability of natural phenolic antioxidants, they cannot meet the needs of large-scale industrial production of food. Synthetic phenolic antioxidants have more excellent antioxidant properties and lower cost than natural phenolic antioxidants [10]. Therefore, in the food-production process, the application of synthetic phenolic antioxidants is more common.

### 1.2. Physicochemical Properties and Hazards of Phenolic Antioxidants

Among the synthetic phenolic antioxidants, tert-butylhydroquinone (TBHQ) is the most commonly used in food due to its strong antioxidant capacity; it is 5–7 times [11] more effective than other antioxidants such as BHA, BHT, and PG. The structural formula is shown in Table 1, TBHQ is a white crystalline powder with a melting point of 126.5–128.5 °C and a boiling point of 291.4 °C. It is highly soluble in ethanol and fats, and moderately soluble in water. TBHQ is chemically stable under heat, but can form a pink product in alkaline conditions.

While TBHQ is widely used in food preservation, its overuse may pose health risks. Studies suggest that excessive TBHQ intake can lead to DNA damage, carcinogenesis, and adverse effects on liver and reproductive health [12,13,14,15,16,17]. Hence, monitoring the levels of phenolic antioxidants in food is essential to prevent excessive consumption.

### 1.3. Regulations and Limits for Phenolic Antioxidants

The hygienic standard for the use of food additives in China ‘GB 2760-1996’ [18] clearly stipulates the usage and dosage of phenolic antioxidants in food. TBHQ is suitable for animal and vegetable fat and fat-rich food, especially for vegetable oil. It is the preferred antioxidant for salad oil, blend oil and high cooking oil. It can be used for edible oils, fried foods, dried fish products, biscuits, instant noodles, boiled rice, canned dried fruits and cured meat products. The upper limit of its use is 200 mg/kg. It is generally recommended to use 0.01~0.02% of the total amount of oil. The dosage of BHA in food is generally not more than 100 mg/kg [19].

Internationally, the maximum allowable use of TBHQ approved by the US Food and Drug Administration (FDA) is 0.02%, and the combined dose is less than 15 mg/kg body weight/day. BHA should not exceed 200 mg/kg, and PG should be limited to the necessary amount for ‘maintaining the quality and taste of normal food’, and the maximum amount should not exceed 0.02% of the total amount. The use of these phenolic antioxidants is more stringent in Japan and the EU. In 2004, Japan completely banned the use of TBHQ in food. TBHQ was detected in seven batches of food exported from China to Japan in 2004, and the related goods were returned or destroyed. In 2004, the EU confirmed the intake standard of TBHQ less than 0.7 mg/kg body weight/day. The European Commission 2018/1481 regulation limits the highest content of TBHQ in different food categories (25–400 mg/kg).

## 2. Research on Analytical Methods of Phenolic Antioxidants

At present, there are many detection and analysis methods for phenolic antioxidants, and each has its own advantages. Analysis methods can be divided into two categories: traditional detection methods and new detection methods [20,21,22,23].

### 2.1. Research Overview of Traditional Detection Methods

Traditional analytical techniques for phenolic antioxidant detection primarily encompass colorimetry, spectrophotometry, gas chromatography (GC) and high-performance liquid chromatography (HPLC) [24].

Specifically, colorimetric and spectrophotometric methods have been widely employed for quantifying phenolic antioxidants in food matrices. A representative example is the determination of butylated hydroxyanisole (BHA), where BHA reacts with 2,6-dichloroquinodichloroimine in a borax buffer to form a blue-colored complex. The BHA concentration can then be quantified by measuring the solution’s absorbance at 620 nm or visually assessing the color intensity against standard references [25,26]. While these methods offer advantages in terms of instrumentation simplicity and applicability in routine laboratory settings, their limited sensitivity (typically in the micromolar range) and susceptibility to matrix interference often result in insufficient accuracy for precise quantification, particularly in complex food samples containing structurally similar phenolic compounds.

Gas chromatography is a technology with the main purpose of detecting the separation and determination of substances. According to the difference of boiling point, polarity and adsorption properties of each substance, the separation of the mixture is realized, and then the content of the target substance is measured [27,28]. The analysis results of this method are accurate and the separation speed is fast, which can meet the requirements of large-scale sampling analysis. However, the sample needs to be pretreated before gas chromatography detection, the process is cumbersome, and the detection requires professional personnel to operate [29,30]. This method is also commonly used in Chinese national standards to detect the content of phenolic antioxidants such as TBHQ, BHA and BHT, such as ‘Determination of tert-butylhydroquinone (TBHQ) in edible vegetable oil’ (GB/T 21512-008) [31] and ‘Determination of tert-butylhydroxyanisole (BHA) and 2,6-di-tert-butyl-p-cresol (BHT) in food’ (GB/T5009.30-2003) [32]. In addition, Xu et al. [31] successfully achieved the simultaneous determination of TBHQ and its transformation products (TBBQ, MAHQ) in fat and oil by gas chromatography–mass spectrometry (GC-MS). Through this method, the initial addition amount of TBHQ can be accurately evaluated, and the recovery rate is more than 90%. It has the potential to accurately identify more TBHQ conversion products in further studies, thereby improving the recovery rate.

High-performance liquid chromatography (HPLC) is a method that uses a single solvent or a mixed solvent as the mobile phase. The mixture is pumped into a chromatographic column containing a stationary phase through a high-pressure infusion system, separated in a chromatographic column, and finally sent to a detector for detection to achieve sample analysis [33,34,35]. This method has played an important role in the field of analysis and detection since its emergence in the late 1960s. Park et al. [36] developed a high-performance liquid chromatography (HPLC) method combined with diode array detector (DAD) for simultaneous detection of 16 phenolic compounds found in the edible aboveground parts of *G. littoralis*. The method uses a gradient elution technique to gradually convert the solvent system from water/acetonitrile to water/methanol. In addition, the method was fully validated, and its linearity, detection limit, quantitative limit, precision, accuracy and recovery rate were evaluated. The results were satisfactory. Finally, the method was applied to the quantitative analysis of phenolic compounds in various G. littoralis samples under different organs, extraction solvents and processing conditions. However, the equipment of liquid chromatography is expensive, the operation cost is high, the analysis time is long and it needs professional training to operate skillfully [35].

### 2.2. Research Overview of New Detection Methods

The new detection methods mainly include capillary electrophoresis, fluorescence spectroscopy and electrochemical analysis [37,38,39].

Capillary electrophoresis is a new type of liquid phase separation and analysis technology, which takes the capillary tube as the separation channel, the high-voltage direct current electric field as the driving force and various characteristics (size, charge, affinity, polarity, etc.) of the analyte as the separation basis [40]. Over the past few years, a more applicable micellar electrokinetic capillary chromatography (MECC) technique has been developed based on this technology. This technology perfectly combines the advantages of high-performance liquid chromatography and capillary electrophoresis, and has the characteristics of high column efficiency, strong selectivity, fast analysis speed, low sample consumption and high degree of automation. Hsi-Ya Huang et al. used polystyrene-divinylbenzene-lauryl methacrylate as capillary stationary phase to analyze five common phenolic antioxidants in edible oil. The analytical method has good reproducibility [41]. However, this technology also has many shortcomings: (1) the reproducibility of sample migration time is poor, so the injection accuracy and detection sensitivity are worse than high-performance liquid chromatography; (2) The capillary diameter is small, resulting in a too short optical path, and the sensitivity of UV absorption spectrum detection results is low. (3) The capillary electroosmotic effect will change due to the difference in the composition of the mixture, which will affect the reproducibility of the separation.

The principle of fluorescence spectroscopy is that some substances will emit light longer than the incident light wavelength after being irradiated by a certain wavelength of light. If this time is relatively short, this light is called fluorescence [42,43,44]. Different substances have different fluorescence spectra due to different molecular structures. Based on this, qualitative and quantitative analysis of substances can be carried out. The fluorescence spectrum can be divided into an emission spectrum and an excitation spectrum. Fluorescence spectroscopy has many advantages, such as high detection sensitivity and strong selectivity [45,46,47].

Electrochemical analysis is an analytical method based on electrochemical principles and techniques. This method can convert the electrochemical properties of the analyte in the solution into intuitive electrical signals (such as current, voltage, and impedance), and determine the content of the analyte by the measured electrical signal intensity [48,49,50,51]. Electrochemical analysis has the advantages of being fast and convenient and having high selectivity, high sensitivity and a low detection limit. It has become a research hotspot in recent years and is widely used in biomedical analysis, environmental monitoring, food analysis and other fields [52,53,54,55]. In addition, due to the good electrochemical activity of phenolic antioxidants, many researchers have used electrochemical analysis methods to quantitatively detect them. As early as in 2000, Romani et al. used differential pulse voltammetry and high-performance liquid chromatography to quantitatively detect phenols in fruits and vegetables, which proved that the electrochemical method had analytical detection performance that matched high-performance liquid chromatography [56]. In addition, Tang et al. [57] used Co nanoparticles derived from a metal–organic framework (ZIF-67) and nitrogen-doped carbon nanotube composites to modify glassy carbon electrodes for electrochemical detection of the TBHQ in edible oil. The combination of cobalt nanoparticles and nitrogen-doped carbon nanotubes not only improves the conductivity, but also enhances the catalytic activity of the electrode, thereby improving the detection sensitivity and stability of TBHQ. This method can quantitatively detect the content of TBHQ in the concentration range of 0.05~80.00 μmol/L. In addition, a large number of studies have shown that the detection results of antioxidants by electrochemical methods are accurate and reliable, and have good selectivity. At the same time, the detection procedure is simple and easy, and the miniaturized electrochemical sensor can also meet the needs of on-site detection. Therefore, some scholars believe that it can be used as an effective way to replace high-performance liquid chromatography and has great market-application prospects [58,59,60].

## 3. Research Progress of Electrochemical Sensors in Phenolic Antioxidants

### 3.1. The Basic Principle of Electrochemical Sensors

**Figure 1:**
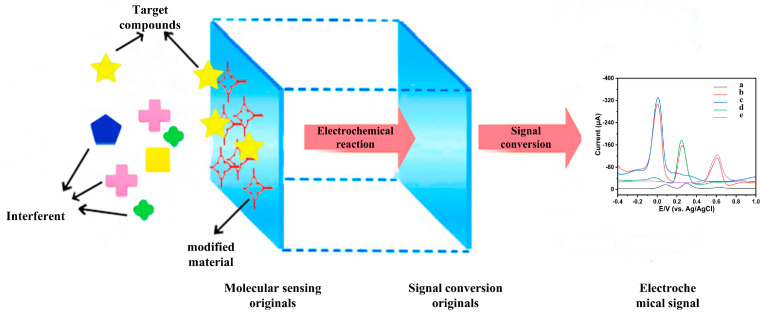
Basic schematic diagram of an electrochemical sensor.

The basic principle of electrochemical sensors is to use the electrochemical reaction of the target detection object on the surface of the working electrode or the electrode-sensitive material to convert the chemical signal into an electrical signal to achieve the detection of the target. The electrochemical sensor is mainly composed of two parts: a molecular sensing element and a signal conversion element [61,62]. The specific structure is shown in Figure 1. Molecular sensing elements are mainly composed of functional materials with high selectivity with respect to the analyte. The function of the signal-conversion unit is to convert the chemical signal measured on the molecular sensing element into an electrical signal, and further analyze and process the results, and finally obtain the analysis results of the object to be tested. Electrochemical sensors can be roughly divided into the following types according to the converted electrical signals: potential sensors, current sensors (amperometric and voltammetric sensors) and impedance sensors (resistive and capacitive sensors) [63,64,65,66].

### 3.2. Research Progress of Electrochemical Sensors in Phenolic Antioxidants

Phenolic antioxidants (such as TBHQ, BHA, BHT and PG) contain benzene ring structure and phenolic hydroxyl groups. In electrochemical detection, the phenolic hydroxyl group is converted into a ketone group by oxidation reaction, which involves electron transfer [67]. The electrochemical workstation can record the intensity of the oxidation peak, which is related to the concentration of the phenolic antioxidant. The oxidation process of the phenolic hydroxyl group includes several steps: first, the hydrogen atom in the phenolic hydroxyl group is protonated and lost to form a phenolic oxygen radical intermediate; then, the phenoxy radical loses an electron through a single electron-transfer mechanism to generate an unstable intermediate state; finally, the intermediate state continues to react, eventually forming a ketone group. The oxidation of phenolic hydroxyl groups can be induced by applying different voltage signals (such as cyclic voltammetry or differential pulse voltammetry) on an electrochemical workstation. When the voltage reaches the oxidation potential, the phenolic hydroxyl groups are oxidized to ketone groups, accompanied by electron transfer and current changes. Since the current generated during the oxidation process is proportional to the concentration of phenolic compounds, the content of phenolic antioxidants in the sample can be quantitatively analyzed by measuring the intensity of the oxidation peak.

Because phenolic antioxidants have significant electrochemical redox properties, they can be directly detected by commonly used commercial electrodes (glassy carbon electrode, screen-printed electrode and carbon paste electrode). However, the detection effect is not ideal, such as weak electrical signal, high detection limit and poor selectivity [68]. Modification of nanomaterials on the surface of these electrodes can significantly improve the sensing performance. At present, the electrode-modification materials mainly include metal nanomaterials, metal oxide nanomaterials, carbon nanomaterials and other nanocomposites [69,70,71,72,73,74,75].

Metal nanomaterials have the advantages of simple synthesis, easy surface functionalization, and enhanced electron-transfer process. They are often used as modified materials for electrochemical sensors, which can improve the diffusion of electroactive substances and improve selectivity [76,77,78,79]. For example, Motia et al. made a molecularly imprinted electrochemical sensor using gold nanoparticle-coated chitosan for the quantitative detection of phenolic antioxidant BHA in food. The introduction of gold nanoparticles further improved the detection limit of the sensor [80]. In addition, Guan et al. [81] developed a high-performance hybrid magnetic nanocomposite with enhanced peroxidase-like activity for electrochemical detection of TBHQ in edible oil. The material embedded AuNPs and magnetic Fe_3_O_4_ nanoparticles into the organic framework by a simple self-assembly method to prepare Fe_3_O_4_@Au/MOF nanocomposites. The improvement of the electrochemical performance of the composite is mainly attributed to its enhanced peroxidase-like activity, which enables Fe_3_O_4_@Au/MOF to effectively catalyze hydrogen peroxide (H_2_O_2_) to generate oxidative radicals and promote the redox reaction of TBHQ, thereby achieving accurate quantitative detection. The experimental results show that the detection limit (S/N = 3) of Fe_3_O_4_@Au/MOF is 0.3323 μM in the range of 1~100 μM of TBHQ concentration. The nanocomposite exhibits excellent stability, repeatability, reproducibility and sensitivity in real samples, demonstrating its potential and practical application value as an electrochemical sensor in the analysis of antioxidants in edible oils.

Metal oxide nanomaterials can obtain more excellent analytical performance by adjusting the particle size, morphology and specific surface area [62]. For example, Zheng et al. [82] successfully prepared heterostructured TiO_2_/Co nanoparticles/nitrogen-doped carbon nanotube (TiO_2_/Co/NCNT) composites by calcining ZIF-67@TiO_2_ at high temperature in Ar/H_2_ atmosphere. The preparation process of the material highlights the key role of TiO_2_ in structure formation and performance improvement. The obtained TiO_2_/Co/NCNT composites exhibit a dodecahedron-shaped box structure with twisted carbon nanotubes covered on the surface. These unique structures provide abundant active sites for electrochemical reactions. TiO_2_ not only enhances the electron-transfer ability in the composites, but also forms a variety of synergistic effects through the interaction with Co, which further improves the electrochemical performance of the composites. It is these structural features and synergistic effects that effectively promote the electrochemical determination of TBHQ, making the material exhibit high sensitivity and stability in the detection of TBHQ.

The application of carbon nanomaterials in the field of electrochemical sensors is mainly to increase the effective area of the electrode, enhance the electron transfer between the electrode and the analyte, and enhance the electrocatalysis [83,84,85]. Feng et al. [86] prepared porous zirconium-based metal–organic framework (NH_2_-UiO-66) and functionalized carbon black (FCB) composites by the one-pot method, and developed a new electrochemical sensor based on this. The sensor used NH_2_-UiO-66@FCB modified glassy carbon electrode (NH_2_-UiO-66@FCB/GCE) to realize the simultaneous detection of butyl hydroxy lettuce (BHA) and tert-butyl hydroquinone (TBHQ). Carbon black plays a crucial role in the preparation of composite materials. As a conductive material, functionalized carbon black significantly enhances the electron-transport capacity of the electrode and promotes the electrochemical reaction. Characterization techniques such as scanning electron microscopy, X-ray diffraction, Fourier transform infrared spectroscopy and X-ray photoelectron spectroscopy showed that the combination of functionalized carbon black and NH_2_-UiO-66 improved the structural stability and surface activity of the material. The electrochemical test results show that the NH2-UiO-66@FCB/GCE sensor has excellent electrochemical behavior. Under the optimized experimental conditions, the linear detection ranges of TBHQ and BHA were 0.12–460 μM and 0.7–420 μM, respectively, and the detection limits were 0.072 μM and 0.29 μM, respectively. In addition, the addition of carbon black not only improves the conductivity of the sensor, but also improves its selectivity, repeatability and stability, which enables the sensor to be successfully applied to the simultaneous detection of TBHQ and BHA in food samples. In addition, Balram et al. [87] successfully prepared hybrid nanomaterials of metal oxides and carbon materials by embedding surface functionalized carbon black (FCB) into Co_3_O_4_ nanorods by the sonochemical method. Highly porous Co_3_O_4_ nanorods were synthesized by the oxalic acid-mediated co-precipitation method, and carbon black was chemically oxidized by acid treatment to improve its surface properties. The obtained hybrid nanocomposite-modified electrode exhibits remarkable electrochemical performance. In the detection of TBHQ, the modified electrode showed a high sensitivity of 7.94 μA μM^−1^ cm^−2^, an ultra-low detection limit of 1 nM and a limit of quantitation of 5 nM. In addition, the potential application value and practicability of the electrochemical sensor in TBHQ detection were verified by its application in actual samples such as tallow, peanut oil and lake water.

MOFs have shown great potential in electrochemical sensors for phenolic antioxidants due to their unique characteristics such as high porosity, easy synthesis, adjustable pore size and surface functionalization [88]. The redox active metal center of MOF can promote charge transfer and enhance the sensitivity and selectivity of the sensor through reversible adsorption and desorption of ions. Its customized structure makes it have significant advantages in improving the performance of electrochemical sensors and it has become a promising material candidate in this field [89]. Aldoori et al. [90] combined the high catalytic activity of platinum nanoparticles with the porous structure and redox properties of MOF, and the synergistic effect of the two significantly improved the performance of electrochemical sensors in the efficient determination of BHA. Platinum nanoparticles play a key role in improving the reaction speed and catalytic activity, while the high surface area and electrochemical activity of MOF improve the sensitivity and selectivity of the sensor, ensuring the accurate detection of butylated hydroxyanisole. The PtN/FE-MOF/GCE sensor exhibits a linear response range from 6.0 × 10^−8^ M to 5.4 × 10^−5^ M with a detection limit of 9.4 × 10^−9^ M. Through the analysis of BHA in potato chip samples, the effectiveness of the sensor in practical applications was further proved.

## 4. Research Progress of Metal Porphyrin Nanocomposites in the Field of Electrochemical Sensing

**Scheme 1:**
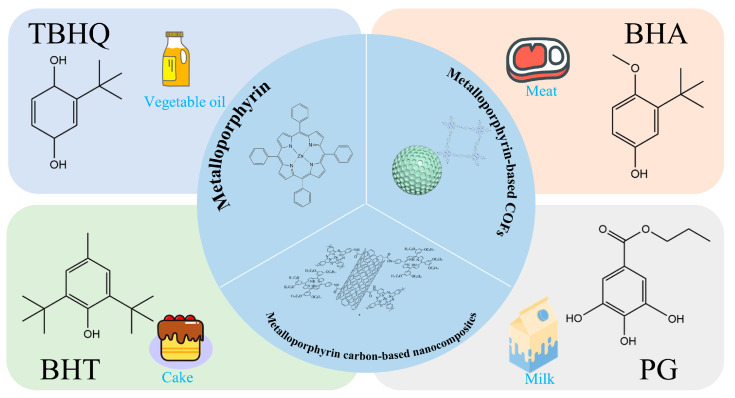
Diagram of metal porphyrin-based electrochemical sensor for the detection of phenolic antioxidants in food.

Significant progress has been made in the application of metal porphyrins and their derivatives in the field of electrochemical sensing, including three types of porphyrin-based sensors (Scheme 1). Firstly, metalloporphyrins have been widely used in electrochemical sensors due to their excellent electron conduction and catalytic properties. Secondly, the metal porphyrin-based COF enhances the performance of the sensor due to its unique structure, high surface area and adjustability, especially in terms of sensitivity and selectivity. Finally, the combination of metal porphyrins and carbon-based nanomaterials further improves the stability and response speed of the sensor. In summary, these metal porphyrin-based materials exhibit excellent performance in the field of electrochemical sensing, providing strong technical support for environmental monitoring, food safety and other fields.

### 4.1. Research Progress of Metalloporphyrins in the Field of Electrochemical Sensing

**Figure 2:**
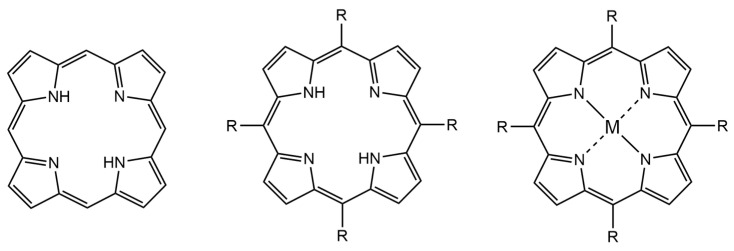
Molecular structures of porphines, porphyrins and metalloporphyrins.

Metalloporphyrins are a class of aromatic heterocyclic compounds with a conjugated macrocyclic structure. Their parent structure is porphin. Porphin is an 18π-electron conjugated system bridged by four pyrrole rings and four methines [91,92,93,94]. The structure is shown in Figure 2, Metalloporphyrin has a stable coordination structure, which can provide a stable coordination environment for the metal core coordinated with it, which provides the possibility for its practical application [95]. At the same time, metalloporphyrin is the catalytic active center of oxidoreductase, which enables it to participate in the redox reaction and then participate in the multi-electron catalytic process. In addition, metalloporphyrins have multifunctional derivative structures, and the periphery and metal core of their macrocyclic conjugated systems can be regulated by various chemical modifications [96]. This modification can not only change the electronic structure of metalloporphyrins, but also significantly affect their photoelectric properties and chemical stability [97]. Through the design and optimization of these modifications, the performance of metal porphyrins can be precisely controlled to show different advantages in different applications. For example, by adjusting the type of metal center or the structure of the peripheral ligand, the conductivity and selectivity of the electrochemical sensor can be improved, thereby improving the response speed and sensitivity of the sensor. Therefore, metalloporphyrins are ideal materials for the preparation of efficient electrochemical sensors due to their excellent photoelectric properties and rich structural diversity.

The working electrode is the most common electrochemical sensor molecular sensing unit. Due to the poor detection performance of the bare electrode, it is not enough to meet the needs of daily detection. Therefore, special composite materials need to be modified on the electrode surface to enhance its detection performance [98,99,100,101]. Metalloporphyrin is an excellent electron donor, and its special structural adjustability and electrocatalytic activity lay the foundation for its successful application in the field of electrochemical sensing. At present, the application of metal porphyrins in the field of electrochemical sensing is mainly divided into the following categories: (1) as electrocatalysts, (2) as a functional material, (3) as a biomimetic catalyst, etc. [102].

**Figure 3:**
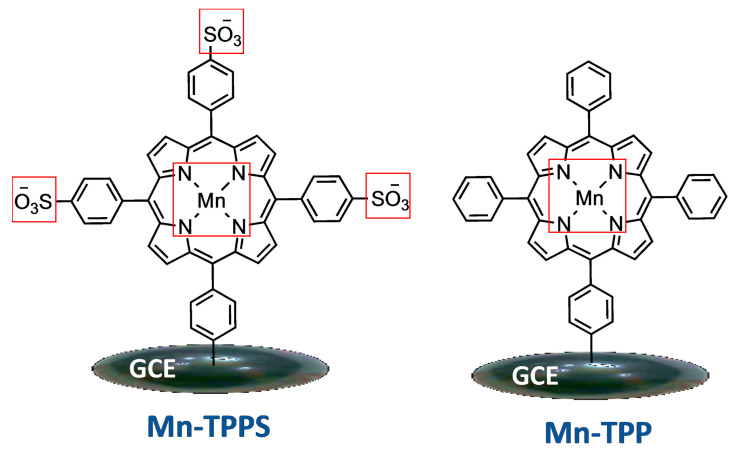
Electroreduction of carbon dioxide to acetate using heterogenized hydrophilic manganese porphyrins [103].

Metal porphyrins based on macrocycles have been widely studied, mainly because metal porphyrin molecules based on macrocycles have better catalytic performance and catalytic selectivity than traditional metal or metal oxide catalysts. As shown in Figure 3, the red box in the figure highlights the modification of the sulfonate group and the central metal of the porphyrin. Abdinejad et al. [103] focused on the key role of manganese porphyrin (Mn-TPP) molecular electrocatalysts in the carbon dioxide-reduction reaction (CO_2_RR). The functionalized manganese tetraphenylporphyrin (Mn-TPP) was immobilized on a glassy carbon electrode (GCE) by the electro-grafting method, achieving a Faraday efficiency of 94%, of which 62% of CO_2_ was converted into acetate. This result shows that manganese porphyrin exhibits excellent catalytic performance in the process of carbon dioxide reduction. Furthermore, Mn-TPP was functionalized by introducing the electron-withdrawing sulfonate group (Mn-TPPS) to adjust its surface properties. These sulfonate groups optimize the surface coverage of the catalyst by electrostatic interaction with water molecules, thereby significantly improving the catalytic efficiency, showing a higher CO_2_ conversion rate than the unfunctionalized Mn-TPP. In the Mn-TPP catalyst without sulfonate group, the main reduction products are carbon monoxide and formate. Under the catalysis of Mn-TPPS, the conversion mechanism has changed, showing a more complex C-C coupling pathway.

Kaur et al. [104] deeply evaluated the electrocatalytic performance of manganese porphyrin (MnPr) and its derivatives in the CO_2_ RR reaction, and especially highlighted the important role of manganese porphyrin as a catalyst. Different MnPr derivatives were designed by axially connecting imidazole, carbene and pyridine groups (MnPr-Im, MnPr-NHC and MnPr-Py, respectively) to optimize their catalytic effects, aiming to efficiently reduce CO_2_ to value-added chemicals while reducing the occurrence of competitive hydrogen evolution reactions (HERs). Based on density functional theory (DFT) calculations, the reaction mechanism and energetic properties of these catalysts in CO_2_ RR were revealed. The results show that MnPr and its derivatives exhibit excellent CO_2_-reduction performance, especially low overpotential and high selectivity during the formation of acetate. Through in-depth analysis of energy distribution, reaction path, scaling relationship, electrode potential and pH value, the excellent reactivity and selectivity of manganese porphyrin derivatives were further elucidated, which made them show extremely high advantages in competition with hydrogen evolution reactions. The results provide strong theoretical support for the design of efficient CO_2_-reduction catalysts, indicating that MnPr and its axially connected derivatives have great potential as electrocatalysts in CO_2_-reduction reactions, with excellent catalytic activity, product selectivity and low overpotential, which provides new ideas and directions for future experimental research and catalyst design. Correa et al. [105] used the self-assembly strategy of metal porphyrin ions with opposite charges to successfully construct a dinuclear site containing a polarized structure, coordinated with Fe (III), Cu (II) or Mn (III) metal ions. The binary porphyrin structure (BIPOS) is composed of metal porphyrin cations carrying pyridine or methylpyridine groups and metal porphyrin anions carrying sulfonyl phenyl groups by conjugation. The BIPOS structures of various metal pairs were studied and compared, including Cu/Fe, Fe/Cu, Cu/Cu, Fe/Fe, Mn/Fe, Fe/Mn and Mn/Mn. In particular, for the Fe/Cu, Fe/Fe and Mn/Fe structures, the electrochemical interaction and catalytic efficiency show a significant enhancement, especially when the metal ions in the BIPOS structure have higher electronegativity (such as Fe) and lower electronegativity metal ions (such as Cu or Mn) are complementary, the catalytic activity is particularly prominent. The results of cyclic voltammetry showed that these highly efficient BIPOS structures exhibited a larger current peak separation (Delta Ep = 0.095–0.125 V) in the catalytic reduction reaction, and the rate constant reached 0.380–0.535 min^−1^/mg in the catalytic reduction process of 4-nitrophenol, which was significantly better than the single metal porphyrin catalyst. In addition, the BIPOS structure also shows excellent advantages in the stability of catalytic performance. For example, Fe/Cu material can maintain high catalytic activity in at least five catalytic cycles, while the Fe/Fe catalyst can also achieve high yield of aniline in nitrobenzene-reduction reactions under mild conditions (such as visible light irradiation, 30 °C, 0.5 mol% catalyst), and 100% yield of aniline can be achieved within 2 h.

**Figure 4:**
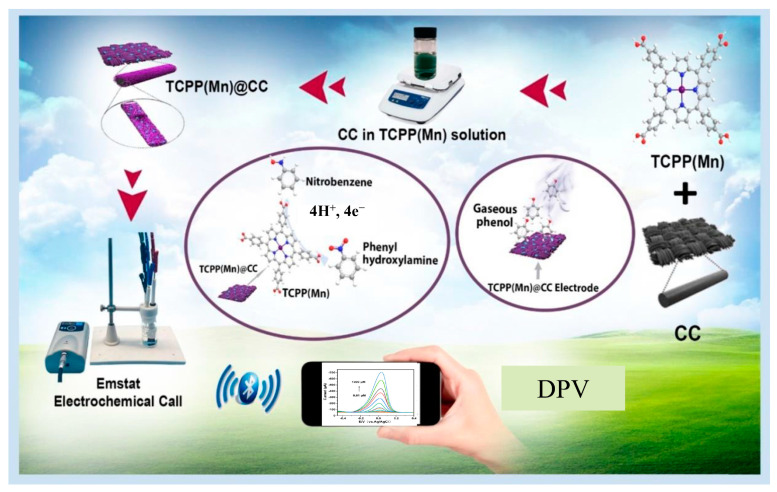
Schematic representation of the synthesis route leading to the TCPP(Mn)@CC electrode and its plausible mechanism toward NB reduction and the detection of gaseous phenol [106].

At the same time, due to the special molecular structure of metal porphyrin, it is also a popular research content as a functional material. As shown in Figure 4 [106], in order to detect nitroaromatics and phenol in water, Andrews Boakye et al. designed and fabricated a portable electrochemical sensor using manganese porphyrin-functionalized carbon cloth material. The results show that the sensor has excellent performance in detecting nitroaromatics and phenol, and the detection limits are 5.9268 × 10^−10^ M and 4.0178 × 10^−10^ M, respectively. At the same time, Liu Xiaoya et al. [107]. found that porphyrins can not only increase the recognition sites of materials, but also enhance their adsorption capacity for phenol and aniline by functionalized reduction of graphene oxide with porphyrin compounds. This improvement significantly improves the detection performance of the sensor for phenol and aniline. This greatly improves the detection performance of the sensor for phenol and aniline. Chu et al. [108] successfully prepared 5,10,15,20-tetra (4-carboxyphenyl) porphyrin (HTCPP)-modified ZnCoO nanospheres (HTCPP-ZnCoO) by the two-step hydrothermal method, and studied the role of porphyrin as a modifier in catalytic activity. Compared with unmodified ZnCoO, HTCPP-ZnCoO exhibited significantly improved peroxidase-like activity and was able to rapidly catalyze the conversion of the substrate 3,3,5,5-tetramethylbenzidine (TMB) to oxTMB in the presence of HO, with obvious blue changes. This catalytic reaction conforms to the Michaelis–Menten kinetic model. The synergistic effect between porphyrin molecules and ZnCoO was further revealed by various techniques such as electrochemistry, fluorescent probes and capture agents, indicating that porphyrins play a key role in promoting electron transfer and superoxide radical formation (O^2^). Based on the nanoperoxidase activity of the HTCPP-ZnCoO catalytic system, a simple colorimetric sensing platform was designed for the detection of HO and cholesterol. The linear range of HO determination was 100–200 μM, and the detection limit was 76.297 μM. The linear range of cholesterol determination was 100–900 μM, and the detection limit was 40.027 μM. This study demonstrates the important role of porphyrin-modified materials in improving nano-catalytic activity and sensing performance.

**Figure 5:**
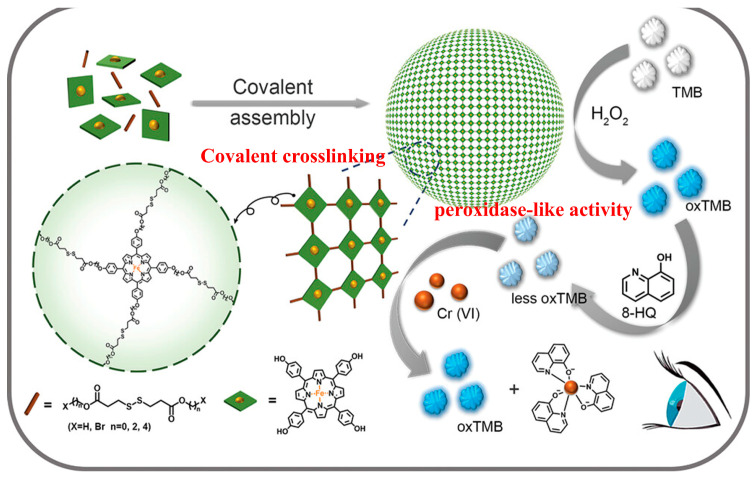
Scheme of covalent assembly CAIPs and Cr(VI) colorimetric detection [109].

In addition, metal porphyrin compounds are the catalytic active centers of horseradish peroxidase, cytochrome c and heme. Therefore, researchers have designed biomimetic enzyme electrochemical sensors based on metal porphyrins. As shown in Figure 5, Xu et al. [109] developed a novel covalently assembled iron porphyrin (CAIP). The material was synthesized by the covalent cross-linking reaction between iron-tetra-(4-hydroxyphenylpropanoid lignin) porphyrin and three cross-linking agents based on 3,3′-dithiodipropionic acid. The results showed that CAIP exhibited a spherical vesicle structure with excellent dimensional stability and enzyme activity. In particular, CAIP exhibited significant peroxidase-like activity under the action of hydrogen peroxide (H_2_O_2_), which could effectively catalyze the oxidation of 3,3′, 5,5′-tetramethylbenzidine. This highly efficient peroxidase activity makes CAIP have excellent catalytic ability in colorimetric reactions. Therefore, CAIP was further applied to the sensitive and selective detection of Cr (VI) with a detection limit of about 23 nM. The method has been verified by standard addition experiments to prove its feasibility in actual water samples. This study provides new ideas and possibilities for the application of nanomaterials in the field of colorimetric detection.

### 4.2. Research Progress of Metal Porphyrin-Based Covalent Organic Frameworks in the Field of Electrochemical Sensing

**Figure 6:**
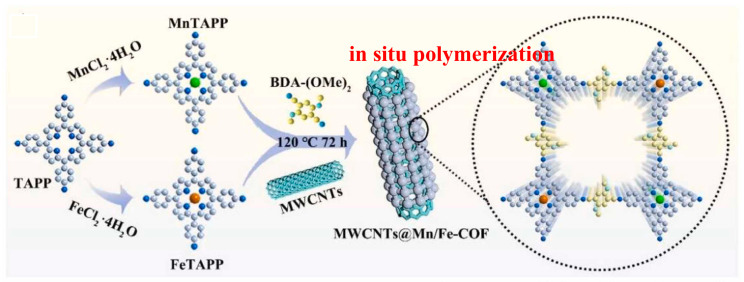
Sketch map of MWCNTs@Mn/Fe-COF synthesis [116].

Covalent organic frameworks (COFs) are a class of two-dimensional or three-dimensional framework materials formed by covalent polymerization between organic monomer compounds [110,111]. As a new type of structural material, COFs have shown great potential in many fields due to their large specific surface area, high porosity, controllable pore structure, strong adsorption performance and abundant functional groups [112,113,114]. Since the first successful synthesis of two-dimensional covalent organic frameworks by Cote et al. in 2005, the variety of COFs has grown rapidly and has gradually entered applications in many fields such as sensing, catalysis, energy storage and filtration [115]. Especially in the sensing field, the highly ordered pore structure and tunable surface functionalization characteristics of COFs make it show excellent performance in gas sensing, chemical sensing and so on. In sensing applications, the high specific surface area and pore structure of COFs provide them with a huge surface area in contact with external molecules, which makes them exhibit extremely high sensitivity in the adsorption and recognition of gas molecules and chemical substances. The functional groups of COFs can interact with specific target molecules, thereby enhancing their selectivity and responsiveness. More importantly, the structure of COF materials can be adjusted by chemical design to achieve accurate identification of different types of gases or chemicals. For example, some COFs can detect low concentrations of harmful gases such as ammonia or carbon monoxide in gas sensors, and have fast response and reversible characteristics, which make them have important application value in environmental monitoring and safety detection. In addition, the stability and reproducibility of COFs make them maintain high sensing performance in long-term operation, which further broadens their application prospects in the sensing field. There are many ways to synthesize COFs, such as imine condensation, boron condensation, solvothermal synthesis and template synthesis. The properties of COFs prepared by different synthesis methods are also quite different. As shown in Figure 6, Bai et al. [116] innovatively prepared a bimetallic porphyrin-based covalent organic framework (COF) composite (MWCNTs@Mn/Fe-COF) by in situ polymerization of manganese porphyrin (MnPor) and iron porphyrin (FePor) on multi-walled carbon nanotubes (MWCNTs). The material not only combines the unique topological structure of porphyrin COF and the excellent conductivity of MWCNTs, but also significantly improves the electrochemical activity. It has become an efficient biomimetic enzyme electrochemical sensor, which can achieve ultra-sensitive detection of trace H_2_O_2_. The special structure of porphyrin COF makes the M-N-4 electrochemical active sites highly independent and provides an efficient electron-transfer pathway. The synergistic effect of the advantages of this structure and the high conductivity of MWCNTs effectively promoted the electrocatalytic reaction. In particular, the introduction of Mn sites forms a self-accelerating redox cycle, which significantly improves the electrocatalytic efficiency, thereby enhancing the sensitivity and selectivity of H_2_O_2_ detection. The sensor based on MWCNTs@Mn/Fe-COF showed a detection limit as low as 2 nM, which reached the standard of ultra-sensitive detection. In addition, the developed sensor has been successfully applied to the detection of trace H_2_O_2_ in real samples, including laboratory ultrasonic cleaners, sterilizers and apple juice. The excellent performance of the sensor provides a reliable tool for the rapid detection of trace H_2_O_2_, demonstrating the great potential of porphyrin COF in the construction of electrochemical sensors.

The electrochemical sensor designed by Ma et al. using the synthesized novel porphyrin-based covalent organic frameworks (PNeCOFs) exhibits excellent detection performance, which indicates that COFs have broad application prospects in the field of electrochemical sensors [117].

Metalloporphyrins are used as building blocks for COFs due to their unique molecular structure, excellent electrochemical properties and easy modification [118]. Therefore, the metal porphyrin-based covalent organic frameworks (MPor-COFs) synthesized based on metal porphyrins have strong structural tunability. Sudhakar et al. found that different substituents do significantly affect the properties of metalloporphyrins [119]. At the same time, due to the unique large π-conjugated structure and metal coordination structure of metalloporphyrins, MPor-COFs can exhibit excellent electrocatalytic activity and selective adsorption capacity for the target [120].

**Figure 7:**
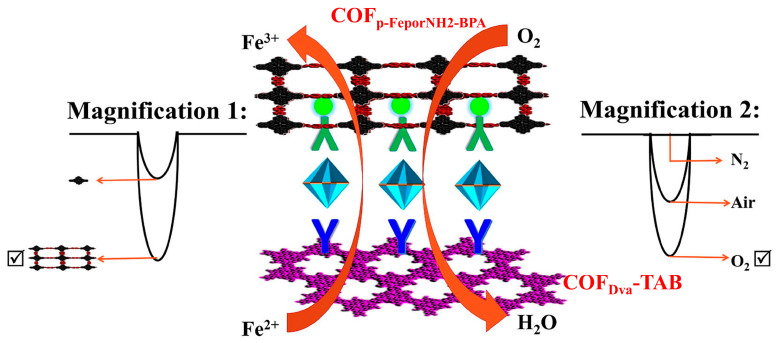
Schematic illustration of the immunosensor construction [107].

At present, the research of metal porphyrin-based covalent organic frameworks mainly focuses on its excellent electrochemical properties, unique porous framework structure and unique electron-adsorption effect. As shown in Figure 7, Xiaoya Liu et al. found that MPor-COFs have a unique large π-conjugated system and abundant recognition sites, which provides theoretical support for selective adsorption and high-sensitivity detection of phenol and aniline in the detection system. The designed P-TP/rGO sensor showed sensitive response to phenol and aniline, and the detection limits were 4.56 nM and 3.01 nM, respectively [107]. Huihui Liang et al. constructed a novel immunosensor using an iron porphyrin covalent organic framework. The sensor utilizes the signal-amplification characteristics of the iron porphyrin covalent organic framework to achieve ultrasensitive detection of enolase, providing a new diagnostic approach for the early diagnosis of lung cancer [121].

### 4.3. The Application of Metal Porphyrin Carbon-Based Nanocomposites in the Field of Electrochemical Sensing

**Figure 8:**
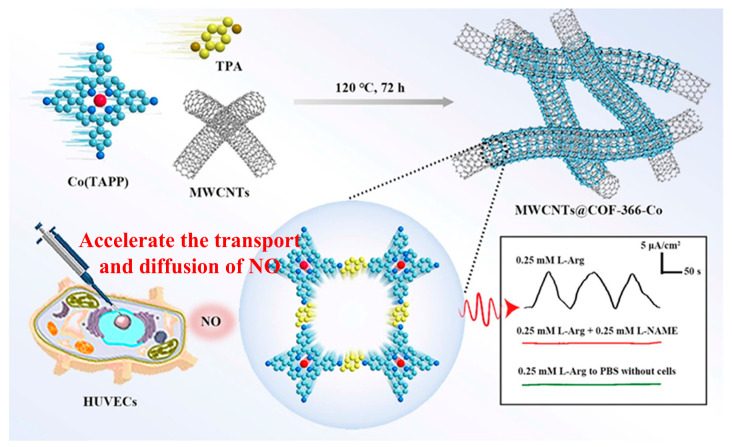
Schematic diagram of the synthesis of MWCNTs@COF-366-Co for real-time detection of NO released from living cells [125].

Multi-walled carbon nanotubes (MWCNTs) and metal porphyrin molecules can form metal porphyrin carbon-based nanocomposites through π-π non-covalent interactions [122,123,124]. Multi-walled carbon nanotubes as a carrier can accelerate the electron transfer between the electrode and the material, which greatly improves the sensitivity of the electrochemical sensor. At the same time, multi-walled carbon nanotubes themselves have certain catalytic properties and can form a synergistic catalytic effect with metal porphyrins. For example, as shown in Figure 8, Bai et al. [125] successfully prepared MWCNTs@COF-366-Co composites by in situ growth of a cobalt porphyrin-based covalent organic framework (COF-366-Co) on the surface of multi-walled carbon nanotubes (MWCNT), and established an ultra-sensitive real-time detection biosensor platform for NO. The design of MWCNTs@COF-366-Co has unique advantages. Firstly, COF-366-Co has highly ordered atomic arrangement and abundant M-N-4 active sites, which significantly improve the electron-transfer efficiency through electrocatalytic action and solve the problems caused by the random arrangement of active sites in traditional materials. These M-N-4 sites exhibit efficient NO-conversion ability in the electrocatalytic process, thus accelerating the transmission and diffusion of NO. Secondly, the high surface area of COF-366-Co provides more space for NO adsorption, and these exposed active sites further optimize the NO transport path. In addition, the synergistic effect of the excellent conductivity of MWCNTs and the atomic-scale periodic structure of COF-366-Co makes the MWCNTs@COF-366-Co electrochemical biosensor exhibit excellent performance in a wide range of NO concentration (from 0.09 nM to 400 μM). Specifically, the sensor has a very high sensitivity (8.9 μA μM^−1^ cm^−2^) and a detection limit as low as 16 nM. This allows the platform to accurately detect extremely low concentrations of NO molecules and achieve efficient and real-time biological monitoring. In order to verify its biosensing performance, MWCNTs@COF-366-Co has been successfully applied to monitor the release of NO in human umbilical vein endothelial cells (HUVECs). Through this platform, NO released by HUVEC can be sensitively detected, demonstrating the great potential of the sensor in the biomedical field.

Srinivas et al. [126] proposed an innovative method to combine the redox activity of heme (iron porphyrin) with multi-walled carbon nanotubes (MWCNTs) to develop a new electrode platform-GCE/MWCNT@HZ-redox. This platform significantly enhanced the activity of iron porphyrin in the electron-transfer process by functionalized MWCNT, and simulated the ability of peroxidase to catalyze the reduction of hydrogen peroxide in a neutral pH solution. The design of the electrode focuses on the use of the redox properties of iron porphyrins to enhance the efficiency of electron transfer on the surface of MWCNT. The biomimetic electrode exhibits excellent electrocatalytic reduction performance of hydrogen peroxide by cyclic voltammetry, which proves the effectiveness of HZ as a redox catalytic center in electrochemical reactions. In terms of electroanalytical performance, the excellent performance of the electrode in hydrogen peroxide sensing was further verified by the combination of batch injection analysis and screen-printed electrodes. The linear concentration range of the electrode is 50–300 μM, the sensitivity is 21 μA μM^−1^ and the detection limit is 220 nM, showing its potential in the detection of hydrogen peroxide. As a bioanalytical application, we successfully achieved in situ monitoring of hydrogen peroxide under reactive oxygen species (ROS) stimulation conditions in HCT-116 colon cancer cells. The electrode platform can detect the change of intracellular H_2_O_2_ in real time and sensitively, which provides a new technical means for the dynamic monitoring of hydrogen peroxide in the process of cell metabolism.

Ma et al. [127] developed a simple and robust non-pyrolysis synthesis method, which successfully constructed a composite material with excellent performance by cleverly in situ coating the electrocatalytically active porphyrin-based thiophene sulfur-site covalent organic polymer (PTS-COP) shell on the core of highly conductive multi-walled carbon nanotubes (MWCNTs). The key design concept is to give the material a strong electrocatalytic activity by accurately anchoring the single-atom Co-N-4 site to the macrocyclic porphyrin structure. The obtained Co-PTS-COPs@MWCNT composite exhibits excellent catalytic performance as an oxygen-reduction reaction (ORR) electrocatalyst. This performance improvement is attributed to two aspects: on the one hand, the 1D core-shell structure (i.e., a multi-walled carbon nanotube core-shell heterostructure with several layers of PTS-COP shells) provides a unique electrocatalytic environment. On the other hand, the atomically anchored Co-N-4 sites in the porphyrin macrocycle and the uniformly dispersed thiophene sulfur sites work together to optimize the electrocatalytic reaction process. It is worth noting that Co-PTS-COPs@MWCNTs have excellent performance in alkaline electrolyte, showing high ORR activity (E-onset is 0.930 V, E^−1/2^ is 0.835 V) and good long-term stability, which is comparable to the most advanced carbon-based electrocatalysts. This study not only shows the synergistic effect between porphyrin and carbon nanotubes, but also provides a new idea for the design of efficient and stable electrocatalytic materials. Basov et al. analyzed a large number of studies on metal porphyrin functionalized carbon nanotubes in recent years, and pointed out that it is a very meaningful topic to use metal porphyrin functionalized carbon nanotubes for electrochemical sensing [128]. A large number of studies have shown that metal porphyrin carbon-based nanocomposites have been successfully applied in the field of electrochemical sensing.

## 5. The Existing Challenges of Electrochemical Sensors Based on Metal Porphyrins

Metalloporphyrin-based electrochemical sensors have broad application prospects, but they still face some challenges in practical applications. The following are the main challenges of metal porphyrin-based electrochemical sensors.

### 5.1. Stability Problem

The stability of metal porphyrin-based electrochemical sensors is a key challenge. The metal porphyrin center may undergo a redox reaction during the electrochemical reaction, resulting in deactivation of the metal center or structural changes, which in turn affects the long-term stability of the sensor. In some cases, metal porphyrin-based sensors may suffer from performance degradation, resulting in reduced response sensitivity. Therefore, it is an urgent problem to improve the stability of metal porphyrin-based electrochemical sensors, especially after long-term use.

### 5.2. Selectivity and Sensitivity

Although the metal porphyrin-based electrochemical sensor has high sensitivity, how to ensure high selectivity in complex samples is still a problem. The structure and functional modification of metal porphyrins have an important influence on their selectivity. With the sensor, it may be difficult to distinguish different target substances in the face of molecules with similar chemical properties. Therefore, optimizing the selectivity of metal porphyrin-based sensors so that they can achieve accurate detection in complex environments is the key to improving their performance.

### 5.3. The Complexity and Cost of Synthesis

The synthesis process of metalloporphyrins usually requires precise control of reaction conditions, including temperature, solvent, catalyst selection and reaction time. These synthetic steps are very complicated and often require high-precision operations, and sometimes even require multiple steps to obtain the final metal porphyrin compound. For example, the synthesis of some metal porphyrins involves the coordination reaction of metal ions with porphyrin ligands, and may need to be carried out in strict anhydrous or inert atmosphere. This high-precision operation not only increases the difficulty of synthesis, but also makes the process difficult to replicate, resulting in a lower yield. Because the synthesis steps of metal porphyrins are cumbersome and involve multiple reaction stages, the time efficiency is low. Each synthesis step may take a long time, especially in the process of multiple purifications and characterizations. In addition, the synthesis process also requires strict reaction control and monitoring to ensure the quality of the final product, which increases the time cost. In large-scale production, especially when it is necessary to respond quickly to market demand, time efficiency becomes a limiting factor. Although the metal porphyrin-based electrochemical sensor has achieved some success on the laboratory scale, it still faces the problem of scalability when it is extended to large-scale production. The current synthesis methods usually work well under small-scale or laboratory conditions, but in large-scale production, problems such as difficult control of reaction conditions, unstable yield, and difficulty in efficient replication of synthesis steps may be encountered.

### 5.4. Reproducibility and the Challenge of Mass Production

The reproducibility and consistency of metal porphyrin-based electrochemical sensors is another challenge. Due to the complex structure of metal porphyrin molecules, subtle differences in the synthesis process may lead to inconsistent performance between different batches of sensors. This poses certain difficulties for large-scale production and commercial applications. Therefore, how to improve the mass production consistency of metal porphyrin-based electrochemical sensors and ensure the stability of each sensor is an urgent problem to be solved.

## 6. Conclusions and Prospects

Metal porphyrin electrochemical sensors have made significant progress in the detection of phenolic antioxidants in food; especially under the modification of metal nanomaterials, metal oxide nanomaterials and carbon nanomaterials, the performance of the sensor has been significantly improved. These sensors exhibit excellent sensitivity, selectivity and stability. Especially after compounding with materials such as multi-walled carbon nanotubes, the electron-transfer efficiency is significantly improved, thereby optimizing the detection performance. As a material with excellent electrochemical performance and catalytic activity, metalloporphyrins have become an important choice to improve the performance of electrochemical sensors. With the continuous advancement of technology, metal porphyrin electrochemical sensors have shown broad application prospects in the rapid and accurate detection of phenolic antioxidants. However, although the metal porphyrin electrochemical sensor has achieved good results in the theoretical and experimental stages, it still faces some challenges in large-scale applications. The synthesis of metalloporphyrins usually requires complex chemical reactions and involves expensive precursor materials and precious metals, resulting in higher costs; at the same time, the multiple steps in the synthesis process and the strict requirements on the reaction conditions affect the time efficiency; in addition, the scalability of metal porphyrin-based electrochemical sensors in large-scale production still faces problems such as inconsistent raw material supply, difficulty in reaction control and consistency in purity. In order to promote the wide application of metal porphyrin electrochemical sensors in practical applications, future research can be optimized from multiple directions: optimizing the synthesis method of metal porphyrins and improving their electron-transfer efficiency and stability, thereby improving the response speed and sensitivity of the sensor; combined with new nanomaterials and conductive substrates, the surface characteristics of the sensor are further improved, and the sensitivity and anti-interference ability are improved. Using the combination of intelligent algorithms and multi-functional sensing platforms, the accuracy of data processing and signal analysis is improved, thereby improving the analysis ability in complex samples. Through the advancement of these research directions, metal porphyrin electrochemical sensors are expected to be more widely used in many fields such as food safety monitoring, environmental monitoring and biological detection, providing efficient and reliable detection solutions.

## Figures and Tables

**Table 1:** Structure and properties of common phenolic antioxidants.

Name	Tert-Butylhydroquinone	Butylated Hydroxyanisole	2,6-Di-tert-butyl-p-cresol	Propyl Gallate
Chemical constitution	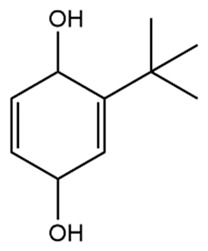	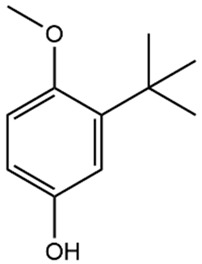	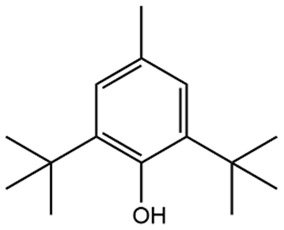	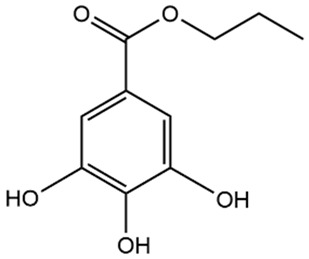
Abbreviation	TBHQ	BHA	BHT	PG
Chemical formula	C_10_H_14_O_2_	C_11_H_16_O_2_	C_15_H_24_O	C_10_H_12_O_5_
Molecular weight	166.217	180.244	220.35	212.21
Cas number	1948-33-0	25013-16-5	128-37-0	121-79-9
Melting point	127~129 °C	48~63 °C	69~71 °C	146~150 °C
Boiling point	291.4 °C	264~270 °C	265 °C	448.6 °C

## Data Availability

No new data were created or analyzed in this study. Data sharing is not applicable to this article.
